# Detection of Very Low-Level Somatic Mosaic *COL4A5* Splicing Variant in Asymptomatic Female Using Droplet Digital PCR

**DOI:** 10.3389/fmed.2022.847056

**Published:** 2022-03-07

**Authors:** Haiyue Deng, Yanqin Zhang, Jie Ding, Fang Wang

**Affiliations:** Department of Pediatrics, Peking University First Hospital, Beijing, China

**Keywords:** Alport syndrome, COL4A5, somatic mosaicism, germline mosaicism, *de novo* disease-causing variant

## Abstract

**Background:**

Alport syndrome is a hereditary glomerulopathy featured by haematuria, proteinuria, and progressive renal failure. X-linked Alport syndrome (XLAS) due to *COL4A5* disease-causing variants is the most common form. In the case of XLAS resulting from 10–18% presumed *de novo COL4A5* disease-causing variants, there are only a few studies for mosaicism in the probands or parents. Very low-level (<1.0%) somatic mosaicism for *COL4A5* disease-causing variants has not been published.

**Materials and Methods:**

Chinese XLAS families with suspected parental mosaicism were enrolled in the present study to evaluate the forms of mosaicism, to offer more appropriate genetic counseling. PCR and direct sequencing were used to detect *COL4A5* disease-causing variants harbored by the affected probands in parental multi-tissue DNAs (peripheral blood, urine sediments, saliva, hair), and droplet digital PCR (ddPCR) was used to quantify the mutant *COL4A5* allelic fractions in parental different samples such as peripheral blood, saliva, and urine sediments.

**Results:**

A Chinese asymptomatic female with suspected somatic and germline mosaicism was enrolled in the present study. She gave birth to two boys with XLAS caused by a hemizygous disease-causing variant c. 2245-1G>A in *COL4A5* (NM_033380) intron 28, whereas this disease-causing variant was not detected in genomic DNA extracted from peripheral blood leukocytes in the woman using Sanger sequencing. She had multiple normal urine test results, and continuous linear immunofluorescence staining of α2 (IV) and α5 (IV) chains of skin tissue. Sanger sequencing demonstrated that *COL4A5* disease-causing variant c. 2245-1G>A was not detected in her genomic DNAs isolated from urine sediments, saliva, and hair roots. Using ddPCR, the wild-type and mutant-type (c.2245-1G>A) *COL4A5* was identified in the female's genomic DNAs isolated from peripheral blood, saliva, and urine sediments. The mutant allelic fractions in these tissues were 0.26% (peripheral blood), 0.73% (saliva), and 1.39% (urine), respectively.

**Conclusions:**

Germline and very low-level somatic mosaicism for a *COL4A5* splicing variant was detected in an asymptomatic female, which highlights that parental mosaicism should be excluded when a *COL4A5* presumed *de novo* disease-causing variant is detected.

## Introduction

Alport syndrome is a hereditary glomerulopathy featured by haematuria, proteinuria, and progressive renal failure. Extrarenal manifestations such as sensorineural hearing loss and ocular abnormalities are often detected. This disease occurs in less than 1:2000 (1) and is caused by disease-causing variants in *COL4A3/A4/A5*, which encode the glomerular basement membrane protein type IV collagen α3–α5 chains, respectively. X-linked Alport syndrome (XLAS) due to *COL4A5* disease-causing variants is the most common form and accounts for 85% (2).

In comparison to male patients with XLAS, female patients have a lower probability of progression to kidney failure at the same age, there is no correlation between their renal outcomes and extrarenal manifestations with COL4A5 genotypes (3). The random X-chromosome inactivation is considered as the reason for the heterogeneity of disease severity in female XLAS cases (4). It is worth noting that low-level (variant allele fraction >1.0%) mosaicism for *COL4A5* disease-causing variants has been observed in rare females with mild phenotypes or even normal urinalysis (5–8), demonstrating that mosaicism influences the disorder manifestations.

Mosaicism, in a diverse range of monogenic disorders, is a biological phenomenon which delineates an individual possessing at least two populations of genetically distinct cells arising from a single zygote (9, 10), and somatic mosaicism is caused by postzygotic *de novo* pathogenic variants (11). Recurrence risk counseling is challenging for a patient with a monogenic disease due to a presumed *de novo* disease-causing variant (12). If this disease-causing variant was inherited from mosaicism in one of the healthy parents, the mosaic parent is at an increased risk of passing on the same disease-causing variant to additional offspring with the risk depending on the proportion of mosaicism within the germ cell progenitors. If this disease-causing variant occurred post-zygotically in the proband, then sibling recurrence risk is very low.

In the case of XLAS resulting from 10–18% presumed *de novo COL4A5* disease-causing variants (13), there are only a few studies for mosaicism in the probands or parents (6, 14–18). To our knowledge, very low-level (variant allele fraction <1.0%) (19) somatic mosaicism for *COL4A5* disease-causing variants has not been published. Here, we describe an asymptomatic female with mutant *COL4A5* allelic fraction (MAF) of 0.26–1.39% in somatic tissues detected by droplet digital PCR (ddPCR).

## Materials and Methods

The clinical data and biological samples of Alport syndrome family in this study were enrolled from a multicentre online hereditary renal diseases registry database and biological sample bank established by Peking University First Hospital in 2012. This study was performed in accordance with the Declaration of Helsinki and approved by the Ethical Committee of Peking University First Hospital (approval number: 2020[72]).

### Patients

Chinese XLAS families with suspected parental mosaicism, who were registered in a multicentre online registry of hereditary kidney diseases, admitted to our hospital during the period of March 2005 to August 2019, and met the inclusion and exclusion criteria were screened in our study. The inclusion criteria were as follows: (1) the probands with XLAS; (2) Sanger sequencing showed *COL4A5* disease-causing variants harbored by the probands did not be detected in their parental DNA samples extracted from peripheral blood leukocytes (3) the probands had at least one sibling with the same *COL4A5* disease-causing variant. Monozygotic twins or patients with unavailable medical records were excluded.

The diagnostic criteria of XLAS were as follows (20): (1) glomerular haematuria with or without proteinuria; (2) negative or intermittent positive immunofluorescence staining for the α5 (IV) chain in the epithelial basement membrane (EBM); (3) *COL4A5* pathogenic variants. XLAS patients were required to meet the criteria (1) along with (2) or (3).

### Research Methods

#### Clinical Data

Patient information, including sex, age, weight, height, family history, clinical findings (age at onset of disease, renal and extrarenal features), and skin and renal biopsy results and genotypes, were reviewed.

#### Genetic Analysis

Genomic DNA from peripheral blood was isolated using FlexiGene DNA Kit (QIAGEN, 51206). Genomic DNA from saliva, hair, and urine sediments were isolated using the QIAamp DNA Micro Kit (Qiagen, 56304).

Multi-tissue DNAs were analyzed by PCR and direct sequencing to detect a *COL4A5* mutation. A 453 bp fragment including *COL4A5* exon 29 was amplified by PCR using a pair of primers (F: 5'-ACCCTGTTTCCAATCCTTCC-3', R: 5'- ATGGCAGCATAGGGTTTCC-3'), which were designed according to the published sequence [UCSC Genome Browser and human GRCh37/hg19 (http://genome.ucsc.edu/)], and analyzed by direct sequencing. The PCR mixture contained 1 μl DNA template, 12.5 μl of 2 × PCR Solution (Takara, RR030A), 1 μl forward and reverse primer (10 pmol/ml), respectively, and 9.5 μl deionized water. Touch-down PCR was used with the following thermal cycling conditions: 94°C for 5 min, 94°C for 30 s, 64°C for 30 s, and 72°C for 45 s. The annealing temperature reduced 1°C every 2 cycles (from 64 to 58°C), 26 cycles at the final annealing temperature of 58°C, with a final extension at 72°C for 10 min, and termination at 4°C.

### Detection and Quantification of Mosaicism

Droplet digital PCR was used to test parental peripheral blood, saliva, and urine sediments DNA to detect mosaicism *via* TD-1^TM^ Digital Droplet^TM^ PCR system (TargetingOne, licensed in China, registration number: 20170025; 20190065; 20192220517). The ddPCR mixture was prepared for each sample with 15 μl 2 × SuperMix, 3 μl probes (final concentration 200 nM each) and primers (final concentration 400 nM each) reaction mix, 2 μl DNA templates, and 10 μl deionized water. The ddPCR thermal profile was as follows: 95°C for 10 min, 40 cycles of 95°C for 30 s and 60°C for 1 min, and 1 cycle of 12°C for 5 min. Data analysis procedure was same as described in previous study (21, 22).

## Results

**Figure 1:**
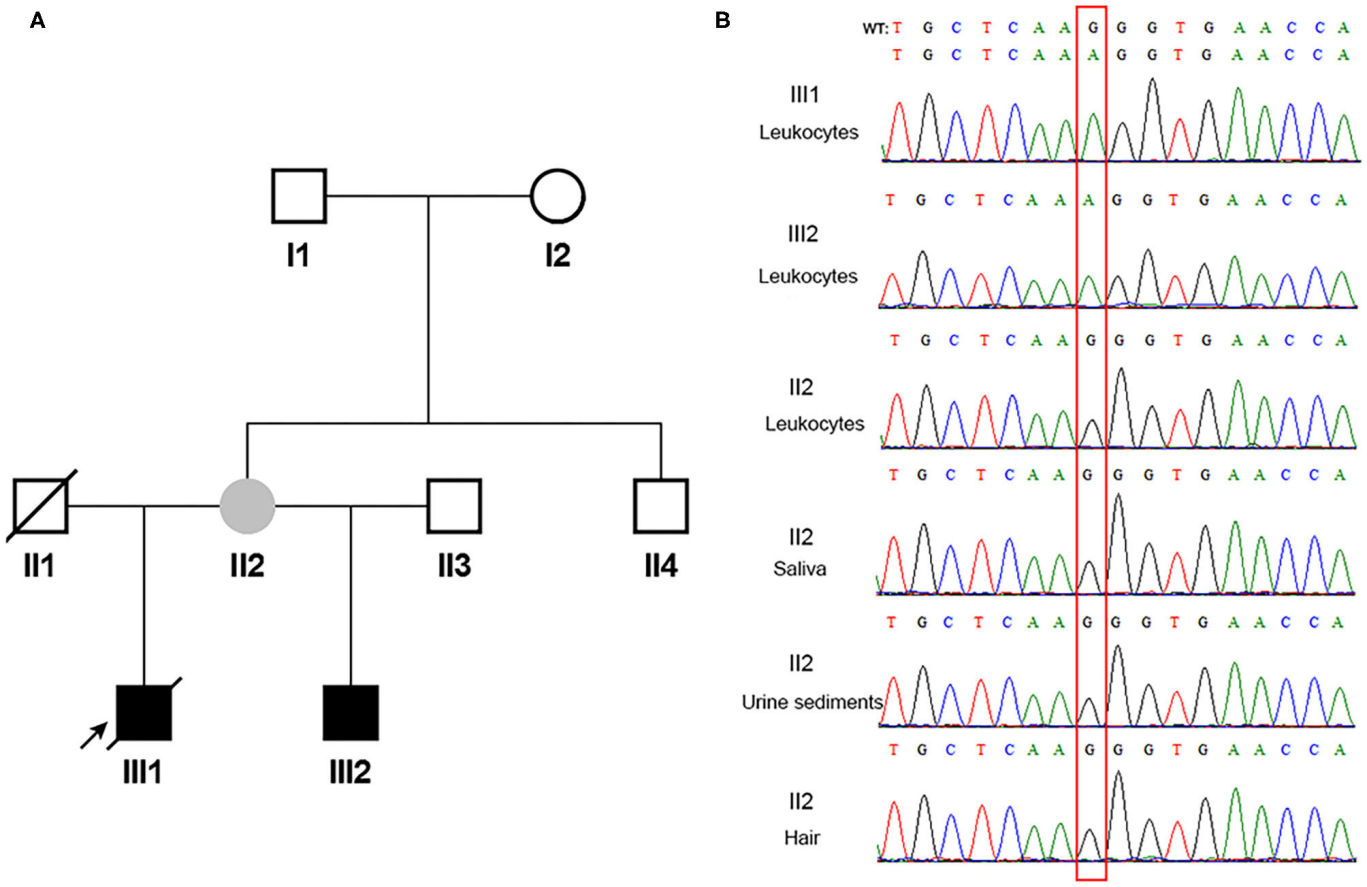
The pedigree and direct sequencing results of multi-tissue in proband's mother. **(A)** The black arrow indicates the proband. The filled black squares indicate the individuals presented with renal phenotypes, and the filled gray circle indicates the suspected mosaicism. **(B)** Direct sequencing results of multi-tissue. The red rectangle indicates c.2245-1 in *COL4A5*. WT, wild type.

According to the inclusion and exclusion criteria, one of 24 patients with *COL4A5* presumed *de novo* disease-causing variants was enrolled. Familial pedigree of the male proband (III1) is shown in Figure 1A. He was found to have haematuria and proteinuria [urinary total protein (UTP): 1.2 g/24h] at the age of 11 years. Renal biopsy, performed at the age of 12 years, indicated mesangial proliferative glomerulonephritis under light microscope, and irregular thickness, lamellated glomerular basement membrane (GBM) under electron microscope. Immunofluorescence staining of skin tissue showed positive for α2 (IV) chain (positive control) and negative for α5 (IV) chain. At the age of 13 years, his serum creatinine was 122 μmol/l. He was diagnosed with chronic kidney disease G5 stage (estimated glomerular filtration rate: 5 ml/min/1.73 m^2^) at the age of 14 years, and died of chronic renal failure at the age of 18 years. The proband's half young brother (III2) was found to have haematuria (110–120/high power field) and proteinuria (UTP: 0.25 g/24h) at the age of 8 years. Immunofluorescence staining of his skin sample showed positive for α2 (IV) chain and negative for α5 (IV) chain. The proband's mother (II2) had multiple normal urine test results, and continuous linear immunofluorescence staining of α2 (IV) and α5 (IV) chains of skin specimen. A splicing variant [*COL4A5* (NM_033380) IVS28: c.2245-1G>A] in a hemizygous state was detected in genomic DNA extracted from peripheral blood leukocytes in the proband and his half young brother by Sanger sequencing, whereas their mother was wild-type (Figure 1B).

Since the asymptomatic mother gave birth to two boys with XLAS, both maternal germline *de novo COL4A5* mutation and somatic and germline postzygotic *COL4A5* mosaicism were highly suspected. Her multi-tissue DNA (saliva, urine sediments, and hair) were extracted and analyzed using PCR and Sanger sequencing. Figure 1B shows that the sites were all wild-type in these tissues.

**Figure 2:**
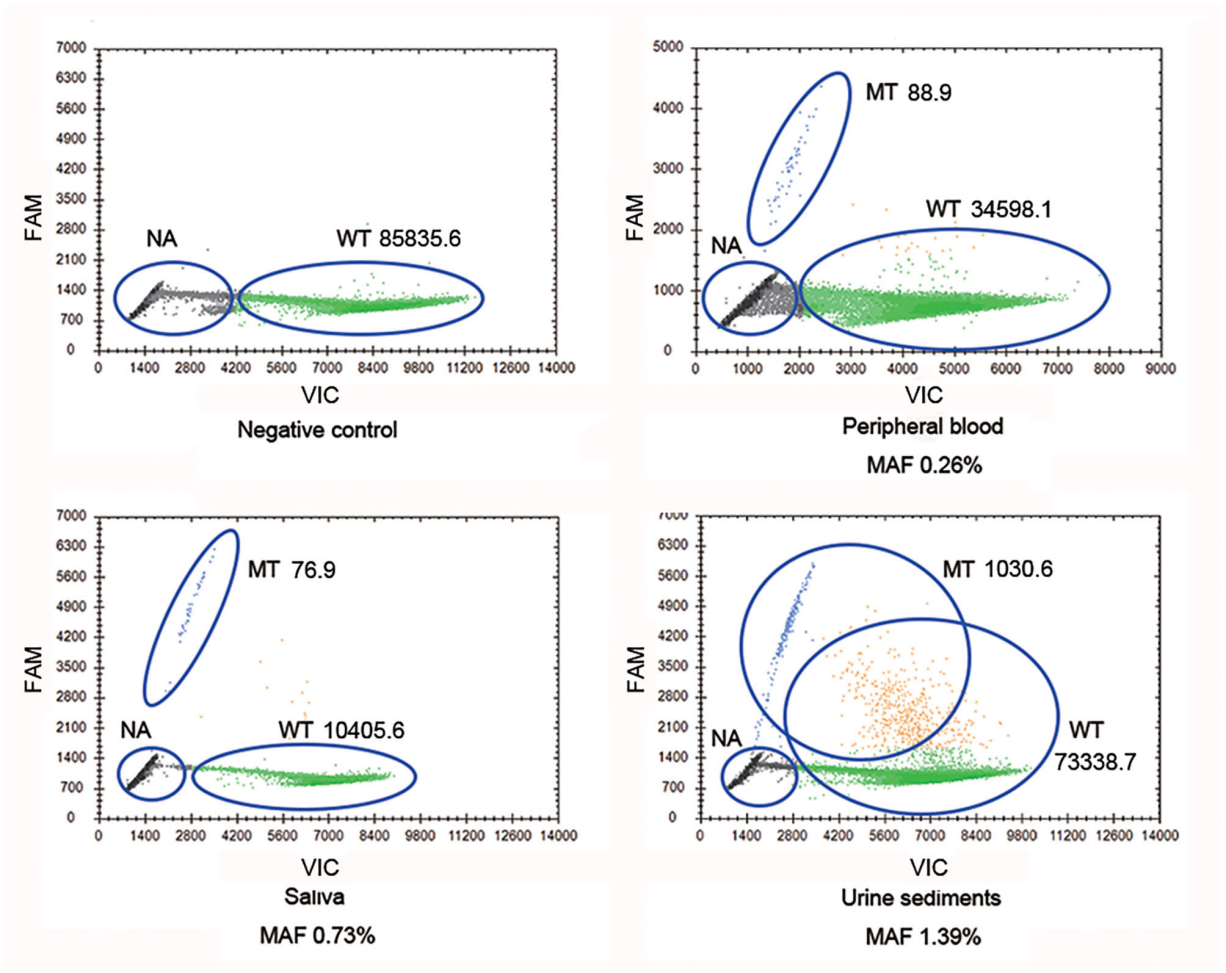
*COL4A5* wild-type and mutant-type (c.2245-1G>A) alleles were identified in multiple samples (blood, saliva, and urine sediments) from proband's mother by droplet digital PCR (ddPCR). WT signals are from droplets containing wild-type alleles, MT signals are from droplets containing mutant alleles, NA signals are from droplets that did not contain target sequences, thus could not be amplified. WT, wild type; MT, mutant type; NA, not available.

Droplet digital PCR, which was carried out since Sanger sequencing, failed to detect mosaic disease-causing variants. It was observed that the mother harbored the wild-type and mutant-type (c.2245-1G>A) *COL4A5* genes, and the MAFs in different tissues were 0.26% (peripheral blood), 0.73% (saliva), and 1.39% (urine sediments), respectively (Figure 2). Therefore, a somatic mosaicism was diagnosed. In addition, germline mosaicism was also diagnosed since she successively gave birth to two affected sons.

## Discussion

In this study, we described a germline and very low-level somatic mosaicism for a *COL4A5* pathogenic splicing variant in an asymptomatic female. This phenomenon was easily overlooked by analyzing Sanger sequencing results, whereas it was indicated through ddPCR method. It highlights that parental mosaicism should be excluded when a *COL4A5 de novo* disease-causing variant is detected.

Mosaicism is a condition where an individual is composed of at least two populations of cells with distinct genotypes. Based on the occurring time and distribution of the mosaic mutation, there are three types of mosaicism (23, 24): somatic (disease-causing variant occurs only in the cells of the body), germline (disease-causing variant occurs only in the germ cells), and somatic combined with germline (disease-causing variant occurs in both germ and body cells). The latter two types of mosaic parents can share the pathogenic variant with their offspring, and the recurrence risk depends on the germline mosaic ratio. As far as XLAS is concerned, parental germline mosaicism or somatic and gonadal mosaicism of *COL4A5* disease-causing variants can transmit the genotypes to the affected offspring. Therefore, it is very essential to identify parental mosaicism earlier to avoid faulty genetic counseling in Alport syndrome families.

At present, a variety of single-gene diseases have been reported that causative genes disease-causing variants mutations could be inherited from mosaic parents (25–27). If a disease-causing variant occurs in the early stage of embryogenesis, a high ratio of disease-causing variant in body and germline cells such as peripheral blood leukocytes could be detected by Sanger sequencing (28, 29). However, Sanger sequencing has limited sensitivity and precision, as only mosaicism with MAF higher than 10% can be effectively identified (30). If a disease-causing variant occurs in the late stage of embryonic cell differentiation, which leads to a low-level of somatic mosaicism, the recognition of mosaicism may be dependent on methods with high analytical sensitivity and precision. Targeted next-generation sequencing can detect a low (MAF in the range of 1–2%) level mosaicism (18). On the other hand, ddPCR can absolutely quantify nucleic acids, the sensitivity can reach 10^−4^ (31, 32), and the detect resolution can be single DNA template. In this study, the proband's mother was not found to be mosaic at the known pathogenic variant site in multi-tissue DNA by Sanger sequencing. Nevertheless, the very low-level MAFs ranging from 0.26 to 1.39% was demonstrated by ddPCR in different tissues. Taking account of her two affected sons, the diagnosis of somatic and germline mosaicism was confirmed. In addition, the fact that she successively gave birth to two affected sons demonstrated her eggs might have a higher fraction of *COL4A5* splicing variant. Since parental germline mosaicism with or without somatic mosaicism can transmit a pathogenic variant from an unaffected parent to their affected offspring, clinicians must consider the possibility of parental mosaicism when facing XLAS patients with presumed de novo disease causing variants, and prenatal or preimplantation genetic testing should be recommended in order to prevent their parents from giving birth to another affected child.

There are few reports about relationship between MAF and phenotypes in XLAS mosaicism patients. For male mosaicism, the lower the MAF, the milder the symptoms. When MAF <50%, the patient may only present with haematuria or even be asymptomatic (18). However, the phenotypes in female mosaicism may be influenced by MAF in kidney, skewed X-chromosome inactivation (33), and modifier genes (34). A previous study showed that an 11-year-old girl had a *COL4A5* disease-causing variant frequency of 22.1% in peripheral blood leukocytes, but she showed moderate proteinuria, which was a severe phenotype for a female. The heterozygous disease-causing variant in modifier gene *COL4A3* may affect disease severity (7). In this study, the proband's mother had multiple normal urinary test results, which could relate to low-level of MAF in the kidney.

In conclusion, we reported a germline and very low-level somatic mosaicism for a *COL4A5* splicing variant in an asymptomatic female, which highlights that parental mosaicism should be excluded when a *COL4A5 de novo* disease-causing variant is identified.

## Data Availability Statement

The original contributions presented in the study are included in the article/supplementary materials, further inquiries can be directed to the corresponding author/s.

## Ethics Statement

The studies involving human participants were reviewed and approved by Ethical Committee of Peking University First Hospital. Written informed consent to participate in this study was provided by the participants' legal guardian/next of kin.

## Author Contributions

HD and FW designed the research study and wrote the manuscript. HD performed the research. HD and YZ analysed the data. FW edited the manuscript. JD provided patient's clinical data, offered advice on designing the research and edited the manuscript. All authors contributed to editorial changes in the manuscript. All authors read and approved the final manuscript.

## Funding

This work was supported by National Key Research and Development Program of China, the registry study of rare diseases in children (No. 2016YFC0901505), National Nature Science Foundation (81070545), Beijing Nature Science Foundation (7102148), and Beijing key laboratory of molecular diagnosis and study on paediatric genetic diseases (BZ0317).

## Conflict of Interest

The authors declare that the research was conducted in the absence of any commercial or financial relationships that could be construed as a potential conflict of interest.

## Publisher's Note

All claims expressed in this article are solely those of the authors and do not necessarily represent those of their affiliated organizations, or those of the publisher, the editors and the reviewers. Any product that may be evaluated in this article, or claim that may be made by its manufacturer, is not guaranteed or endorsed by the publisher.
